# Accuracy of a sequential algorithm based on FIB-4 and ELF to identify high-risk advanced liver fibrosis at the primary care level

**DOI:** 10.1007/s11739-023-03441-2

**Published:** 2023-11-11

**Authors:** Pablo Gabriel-Medina, Roser Ferrer-Costa, Andreea Ciudin, Salvador Augustin, Jesus Rivera-Esteban, J. M. Pericàs, D. M. Selva, Francisco Rodriguez-Frias

**Affiliations:** 1 Clinical Biochemistry Department, Vall d’Hebron University Hospital, 08035 Barcelona, Spain; 2 Biochemistry and Molecular Biology Department, Universitat Autònoma de Barcelona (UAB), 08193 Barcelona, Spain; 3 Clinical Biochemistry Research Team, Vall d’Hebron Institut de Recerca (VHIR), 08035 Barcelona, Spain; 4 Endocrinology and Nutrition Department, Vall d’Hebron University Hospital, 08035 Barcelona, Spain; 5 Diabetes and Metabolism Department, Vall d’Hebron Institut de Recerca (VHIR), Universitat Autònoma de Barcelona (UAB), 08035 Barcelona, Spain; 6 Centro de Investigación Biomédica en Red de Diabetes y Enfermedades Metabólicas Asociadas (CIBERDEM), 28029 Madrid, Spain; 7 Liver Unit, Internal Medicine Department, Vall d’Hebron Institut de Recerca (VHIR), Vall d’Hebron University Hospital, 08035 Barcelona, Spain; 8 Centro de Investigación Biomédica en Red de Enfermedades Hepáticas y Digestivas (CIBEREHD), 28029 Madrid, Spain

**Keywords:** Advanced liver fibrosis, NASH, FIB-4, ELF, Type 2 diabetes, Chronic liver disease

## Abstract

Non-alcoholic fatty liver disease (NAFLD) is the leading cause of chronic liver disease, and liver fibrosis is the strongest predictor of morbimortality. We aimed to assess the performance of a sequential algorithm encompassing the Fibrosis 4 (FIB-4) and Enhanced Liver Fibrosis (ELF) scores for identifying patients at risk of advanced fibrosis. This cross-sectional study included one hospital-based cohort with biopsy-proven NAFLD (*n* = 140) and two primary care cohorts from different clinical settings: Type 2 Diabetes (T2D) follow-up (*n* = 141) and chronic liver disease (CLD) initial study (*n* = 138). Logistic regression analysis was performed to assess liver fibrosis diagnosis models based on FIB-4 and ELF biomarkers. The sequential algorithm retrieved the following accuracy parameters in predicting stages F3–4 in the biopsy-confirmed cohort: sensitivity (85%), specificity (73%), negative predictive value (79%) and positive predictive value (81%). In both T2D and CLD cohorts, a total of 28% of patients were classified as stages F3–4. Furthermore, of all F3–4 classified patients in the T2D cohort, 80% had a diagnosis of liver disease and 44% were referred to secondary care. Likewise, of all F3–4 classified patients in the CLD cohort, 71% had a diagnosis of liver disease and 44% were referred to secondary care. These results suggest the potential utility of this algorithm as a liver fibrosis stratifying tool in primary care, where updating referral protocols to detect high-risk F3–4 is needed. FIB-4 and ELF sequential measurement is an efficient strategy to prioritize patients with high risk of F3–4 in populations with metabolic risk factors.

## Introduction

Chronic liver disease (CLD) is a major cause of mortality globally and leads to a substantial health-care burden [1]. CLD often presents asymptomatically until advanced phases, when liver damage is irreversible and therapy can only slow or stop progression of the disease [2, 3]. Non-alcoholic fatty liver disease (NAFLD) is the leading cause of CLD worldwide, affecting 17–46% of adults in high-income countries [4–6]. Around 20% of patients with NAFLD progress to nonalcoholic steatohepatitis (NASH) with various degrees of fibrosis and eventually cirrhosis and hepatocellular carcinoma [7]. Liver fibrosis is the strongest predictor of clinically meaningful outcomes, including decompensation, cardiovascular and liver-related morbimortality [8–10].

The tools used in the screening of CLD with advanced fibrosis are paramount to designing and implementing efficient, sustainable, and equitable health-care pathways both in the general population at community and primary care centers and special populations at high-risk. NAFLD and metabolic syndrome (MetS) are intimately related entities [11]. Additionally, Type 2 Diabetes Mellitus (T2D) is a well-known risk factor for NAFLD [12]. The prevalence of NAFLD among T2D patients increases to 60–80%, and it has been consistently shown that T2D also acts as a trigger by promoting the progression to NASH and advanced liver fibrosis [5, 13].

The gold standard of NAFLD diagnosis is based on histological assessment by liver biopsy. Liver biopsy is an invasive procedure that can lead to complications [14] and a significant diagnostic error rate [15, 16]. Use of liver biopsy is largely limited to screening liver disease on a large scale. In recent years, noninvasive markers as ^13^C Methacetin Breath Test, which assess microsomal liver function [17], or techniques have been proposed for the screening of liver disease, such as transient elastography (TE) and acoustic radiation force impulse shear wave elastography (ARFI), which can predict and monitoring significant fibrosis from different etiologies [18–20]. TE has proven to be cost-effective for population screening of liver fibrosis [21], but its availability is currently limited in primary care centers and requires certain training for operators [22].

Several serum biomarkers and panels have been developed to detect significant liver fibrosis (equivalent to F2–F3 fibrosis stages in liver biopsy) or advanced liver fibrosis (F3–F4) in NAFLD patients [23, 24]. Fibrosis 4 score (FIB-4) was initially defined to predict significant fibrosis in patients with HIV/HCV coinfection [25]. FIB-4 performs best at excluding advanced fibrosis (with negative predictive values > 90%) and is, therefore, commonly used as a first-line triage to identify patients at low risk of severe fibrosis [26]. Current guidelines by the European Association for the Study of the Liver propose two FIB-4 cut-off points (1.3 and 2.67) to rule out advanced fibrosis according to the age of the patient [27]. However, some reports suggest that FIB-4 accuracy might be impaired amongst patients with T2D [28]. The Enhanced Liver Fibrosis Test (ELF™, Siemens Healthineers, Tarrytown, NY, USA) is a blood panel that combines results for tissue inhibitor of matrix metalloproteinase type 1 (TIMP-1), hyaluronic acid (HA), and aminoterminal propeptide of type III procollagen (PIIINP) into a single score or index. All three markers are involved in hepatic extracellular matrix metabolism (fibrinolysis or fibrinogenesis). Different cut-off values have been described to stratify patients into none to mild fibrosis, moderate fibrosis, and severe fibrosis [29, 30]. The ELF score has shown excellent accuracy for the non‐invasive diagnosis of advanced fibrosis in different cohorts [31]. In addition, The ELF score is able to predict clinical outcomes [26].

Several reports have provided information on the performance of a sequential algorithm including FIB-4 and ELF into routine primary care, where the active participation of general practitioners and physicians who manage patients with metabolic disorders is crucial [32, 33]. There is poor application of these based-on biomarker algorithms in our health system, and their implementation is needed in clinical laboratories to detect patients with high risk of advanced fibrosis. Detecting patients at high risk of advanced fibrosis would strongly facilitate further advanced fibrosis screening. Use of a FIB-4 and ELF sequential algorithm would be especially useful for primary health care, where the knowledge about degree of liver fibrosis is limited [34–36].

We aimed to assess a FIB-4 and ELF algorithm to diagnose liver fibrosis in a NASH cohort and propose its application to stratify the risk of fibrosis in two primary care cohorts with liver-related comorbidities.

## Materials and methods

We performed a cross-sectional study, including consecutive subjects diagnosed with NASH from January 2016 to December 2019 at the Liver Unit of the Vall d’Hebron University Hospital, Barcelona, Spain. The study was conducted according to the Declaration of Helsinki and was approved by the local ethics committee (PR(AG)601/2020). Liver and biochemical samples from patients included in this study were provided by the Vall d’Hebron University Hospital Biobank (PT17/0015/0047), integrated in the Spanish National Biobanks Network, and they were processed following standard operating procedures with the appropriate approval of the ethical and scientific committees. Serum samples were drawn at the same time that liver biopsy was performed (if applicable), as per protocol. All participants had previously signed the informed consent.

### NASH cohort

Inclusion criteria: (a) age > 18 years. (b) NASH diagnosis by liver biopsy.

Exclusion criteria: (a) potentially harmful alcohol consumption (> 30 g/day for men and > 20 g/day for women), (b) other causes of liver disease (viral or autoimmune hepatitis, hereditary hemochromatosis, alcoholic liver disease, liver transplantation, etc.), (c) hepatotoxic drugs, and (d) uncontrolled endocrine diseases (hypothyroidism, hypercortisolism, etc.).

Liver histology was evaluated according to the Clinical Research Network (CRN) NASH criteria [37]: (a) steatosis was scored 0–3 (b) lobular inflammation was scored 0–3 (c) ballooning (marker of cell injury) was scored 0–2 (d) NASH activity score corresponded to the unweighted sum of the scores for steatosis, lobular inflammation and ballooning; finally, (e) fibrosis was staged 0–4. Advanced liver fibrosis was defined as the presence of fibrosis grades 3–4 in the histological evaluation. Furthermore, TE (FibroScan^®^) and ultrasonography measures were performed in the entire cohort. Steatosis was graded as follows: Absent (score 0), echotexture of the liver is normal; mild (score 1), slight and diffuse increase of liver echogenicity with normal visualization of the diaphragm and of the portal vein wall; moderate (score 2), moderate increase of liver echogenicity with slightly impaired appearance of the portal vein wall and the diaphragm; severe (score 3), marked increase of liver echogenicity with poor or no visualization of portal vein wall, diaphragm, and posterior part of the right liver lobe [38]. The grading of fibrosis was obtained using Fibroscan 502 Touch devices (Echosens, Paris, France) equipped with M and XL probes. All measurements were performed by a specialized health-care professional experienced with the procedure. TE measurements were performed under usual and manufacturer standards. A liver stiffness measurement was considered reliable if an interquartile range/median (IQR/M) ratio < 0.30 was achieved, and only examinations with at least 10 individual measurements were deemed valid [27, 39, 40].

### Primary care cohorts

Since Vall d’Hebron Hospital Clinical Laboratories provides clinical analysis service to primary care patients, we decided to include a population of patients that are regularly monitored at the analytical level in our hospital. To simplify and increase the efficiency of requesting analytical tests in a routine care setting, the Catalonian Health System has implemented a set of Primary Care Protocols (PCPs) with multiple tests. Community clinicians can request those PCPs that better suit each clinical situation or diagnostic suspicion. The PCPs considered were T2D annual follow-up and initial study of CLD.

Inclusion criteria: (a) age > 18 years, (b) T2D diagnosis defined according to the American Diabetes Association (ADA) guidelines [41], for the primary care T2D cohort, (c) suspicious liver disease: alcohol-related liver disease, chronic viral hepatitis, non-alcoholic liver steatosis, metabolic syndrome-related liver disease, autoimmune hepatitis, hepatotoxic drugs or CLD of unknown etiology, for the primary care CLD cohort.

Exclusion criteria: uncontrolled endocrine diseases (hypercortisolism, etc.).

These cohorts were included as a real-world study of the degree of liver fibrosis diagnosis, so liver samples were not available.

### Biomarker measurement and noninvasive models of diagnosis of liver fibrosis

Hepatic insulin resistance (IR) was indirectly evaluated using the Homeostatic Model Assessment for Insulin Resistance (HOMA-IR), based on the formula [fasting glucose (mg/dl)*fasting insulin (μU/mL)/405] [42]. A cut-off ≥ 3.02 has been described as a marker of IR in Caucasian population [43]. Patients with T2D on insulin treatment were excluded from the calculation of HOMA-IR.

The FIB-4 score was calculated following the formula: (age [years] × AST [U/L])/(platelets [10^9^/L] × (ALT [U/L])^1/2^) [26], and the ELF score was measured by immunoassay on ADVIA Centaur^®^ analyzers (Siemens Healthcare Diagnostics Inc., Tarrytown, NY, USA) [30].

Different liver fibrosis diagnosis models, non-significant fibrosis (F0–1) and advanced fibrosis (F3–4), were assessed in the biopsy-confirmed NASH cohort according to noninvasive biomarker results, considering liver biopsy as gold standard; namely, FIB-4 alone, ELF alone, FIB-4 and ELF in multivariate analysis, and FIB-4 and ELF in a sequential algorithm. The sequential algorithm involved an initial FIB-4 calculation in all patients. Those with FIB-4 < 1.30 were classified as F0–1 and those with FIB-4 ≥ 2.67 were classified as F3–4. In patients with FIB-4 intermediate values (1.30 ≤ FIB-4 < 2.67), ELF was measured, where ELF < 8.30 indicated F0–1 and ELF ≥ 9.50 indicated F3–4. Patients with intermediate values in ELF test (8.30 ≤ ELF < 9.50) were classified as high risk of F2–4. Additionally, FibroScan measurement was included for high-risk F3–4 fibrosis to complement the noninvasive diagnosis in the sequential algorithm.

The sequential algorithm was assessed in primary care cohorts (T2D and CLD) to estimate the degree of liver fibrosis diagnosis, according to the evidence of liver disease in patients’ medical records (i.e., confirmed by TE, magnetic resonance or ultrasonography findings).

### Statistical analysis

The distribution of data was assessed by the Kolmogorov–Smirnov test. ANOVA and Kruskal–Wallis tests were used to compare quantitative variables which followed a Gaussian distribution or not, respectively. Chi-squared test was used to compare proportions.

Logistic regression and Area Under Curve of Receiver Operator Characteristic (AUC-ROC) analysis were performed to assess liver fibrosis diagnosis models based on noninvasive biomarkers (FIB-4 and ELF). Parameters of diagnosis accuracy where calculated based on cut-offs proposed. All statistical analyses were performed with R-commander (R-UCA package, v.2.6-2).

## Results

### Characteristics of the study cohort

A total of 140 NASH patients fulfilling inclusion criteria were identified. Furthermore, 141 T2D primary care patients and 138 CLD primary care patients were included. Baseline characteristics of the three groups are shown in Table 1. Anthropometric variables (body mass index and waist circumference) were similar between NASH and T2D primary care cohorts and higher than CLD primary care patients, as well as glucose and lipids metabolism parameters (fasting glucose, HOMA-IR, triglycerides, cholesterol LDL and HDL).

**Table 1:** Clinical characteristics in the cohorts studied. Values are mean (standard deviation), number (%) or median (Q1–Q3)

Variable	NASH (*n* = 140)	Primary care T2D (*n* = 141)	Primary care CLD (*n* = 138)	*P* value
Age (years)	59 (10)	57 (10)	56 (11)	0.093
Sex. Female	81 (58%)	71 (50%)	68 (49%)	0.294
BMI (kg/m^2^)	32 (5)	32 (7)	27 (5)	**< 0.001**^**a**^
Waist circumference (cm)	108 (12)	104 (13)	92 (10)	**< 0.001**^**a**^
Fasting glucose (mg/dL)	129 (55)	148 (69)	90 (19)	**< 0.001**^**b**^
HbA1c (%)	6.5 (1.4)	7.5 (1.8)	5.7 (0.6)	**< 0.001**^**b**^
HOMA-IR	7.41 (6.40)	5.41 (4.04)	1.97 (1.07)	**< 0.001**^**a**^
Triglycerides (mg/dL)	153 (113–206)	156 (112–204)	109 (77–153)	**< 0.001**^**a**^
Cholesterol LDL (mg/dL)	116 (37)	116 (43)	137 (37)	**< 0.001**^**a**^
Cholesterol HDL (mg/dL)	49 (12)	48 (12)	55 (15)	**< 0.001**^**a**^
ALT (IU/L)	46 (31–71)	26 (20–40)	24 (21–32)	**< 0.001**^**c**^
AST (IU/L)	42 (29–59)	31 (20–49)	24 (15–33)	**< 0.001**^**b**^
GGT (IU/L)	73 (41–160)	43 (27–71)	29 (19–49)	**< 0.001**^**c**^
FIB-4	1.50 (1.06–2.38)	1.19 (0.85–1.53)	1.29 (0.92–1.78)	**< 0.001**^**c**^
ELF	9.57 (8.90–10.44)	9.66 (9.13–10.23)	9.41 (8.90–10.22)	0.367
Fibroscan	11.75 (9.00–17.08)			
Ultrasonography				
Mild steatosis	63 (45%)			
Moderate steatosis	43 (31%)			
Severe steatosis	34 (24%)			

Bold values indicate statistical significance

*BMI* body mass index, *HOMA-IR* homeostasis model assessment of insulin resistance, *ALT* alanine aminotransferase, *AST* aspartate aminotransferase, *GGT* gamma-glutamyl transpeptidase, *FIB-4* fibrosis 4 score, *ELF* enhanced liver fibrosis

^a^Significant differences between NASH and primary care T2D vs primary care CLD groups

^b^Significant differences between all groups

^c^Significant differences between NASH vs primary care T2D and primary care CLD groups

In the NASH cohort, 66% were diabetics (*n* = 93). T2D treatment approaches included metformin (77%), either as a single treatment (27%) or co-administered with insulin (23%), glucagon-like peptide-1 (GLP-1) analogs (9%), inhibitors of sodium–glucose cotransporter-2 (iSGLT2) (12%), or inhibitors of dipeptidyl peptidase IV (iDPP-IV) (6%); insulin alone (9%), and diet only (14%).

Biopsy, ultrasonography and biomarkers results of NASH cohort are shown in Table 2. The majority of patients had steatosis 0–1 (*n* = 90) by histological study and mild steatosis (*n* = 80) by ultrasonography measure, but only 50 of them had the similar classification. Otherwise, both FIB-4 and ELF biomarkers and TE measures showed increasing values with higher degrees of fibrosis.

**Table 2:** Summary of biopsy findings, ultrasonography measures and FIB-4, ELF and Fibroscan values in the NASH cohort

Biopsy results		Ultrasonography		
Steatosis		Mild steatosis	Moderate steatosis	Severe steatosis
0–1	90 (64%)	50 (36%)	25 (18%)	15 (11%)
2	39 (28%)	10 (7%)	14 (10%)	15 (11%)
3	11 (8%)	3 (2%)	4 (3%)	4 (3%)
NASH activity score				
≤ 3	55 (39%)			
4	40 (29%)			
5	27 (19%)			
≥ 6	18 (13%)			

Fibrosis		FIB-4	ELF	Fibroscan
0–1	38 (27%)	1.04 (0.85–1.36)	8.93 (8.52–9.47)	9.00 (7.90–10.80)
2	23 (17%)	1.50 (1.08–1.82)	9.31 (8.83–9.75)	9.65 (7.88–12.45)
3–4	79 (56%)	1.95 (1.44–3.01)	10.10 (9.22–10.91)	15.00 (10.95–20.90)

Values are number (%) for biopsy and ultrasonography results and median (Q1–Q3) for FIB-4, ELF and Fibroscan measures

In the T2D primary care cohort, treatment approaches included metformin (42%), insulin (7%), GLP-1 analogs (7%), iSGLT2 (9%), iDPP-IV (11%), sulfonylureas (7%) and diet only (17%).

Diagnostic approaches for CLD in the primary care cohort were alcohol-related liver disease (8%), chronic hepatitis C infection (4%), non-alcoholic steatosis (15%), metabolic syndrome-related liver disease [high blood pressure (11%), dyslipidemia (20%), obesity (4%), hyperuricemia (6%) and hypothyroidism (6%)], liver cancer (1%), gastroenterological and liver lithiasis diseases (7%), cardiovascular disease (3%) and others (15%).

### Noninvasive models of diagnosis of liver fibrosis in NASH cohort

Nonsignificant fibrosis (F0–1) and advanced liver fibrosis (F3–4) diagnosis models evaluated in the NASH cohort were: FIB-4 alone, ELF alone, FIB-4 and ELF in multivariate analysis and FIB-4 and ELF sequential algorithm. Summary of data and diagnostic accuracy parameters for all models is shown in Table 3.

**Table 3:** Model comparison for liver fibrosis diagnosis in the whole NASH cohort

	Diagnosis	Cut-off	AUC	IC95%	Sensitivity (%)	IC95%	Specificity (%)	IC95%	PPV (%)	IC95%	NPV (%)	IC95%
FIB-4	F0–1	< 1.3	0.79	0.71–0.86	68	51–83	75	65–83	50	36–64	86	76–93
	F3–4	≥ 2.67	0.80	0.72–0.86	35	25–47	98	91–100	97	82–100	55	45–64
ELF	F0–1	< 8.30	0.76	0.68–0.83	18	8–34	94	88–98	54	25–81	76	67–83
	F3–4	≥ 9.50	0.76	0.68–0.83	68	57–78	70	57–82	75	63–85	63	51–75
Multivariate regression	F0–1	–	0.81	0.73–0.87	61	46–77	82	71–94	56	39–71	85	76–91
	F3–4	–	0.81	0.73–0.87	80	71–89	70	60–81	78	67–86	73	61–84
Algorithm	F0–1	Combined^a^	0.76	0.68–0.83	71	57–85	81	69–94	58	43–72	88	80–90
	F3–4	Combined^b^	0.74	0.66–0.81	85	78–91	73	64–83	81	69–89	79	64–89
	F2–4	Combined^c^	0.62	0.43–0.80	88	65–96	58	43–73	85	66–97	50	36–63
	F ≥ 2	Combined^b,c^	0.76	0.68–0.83	81	72–88	71	54–85	89	81–95	57	41–72
TE	F ≥ 2	≥ 8 kPa	0.78	0.70–0.84	90	83–96	30	16–47	77	68–84	55	31–78

*AUC* area under curve, *PPV* predictive positive value, *NPV* negative predictive value

^a^FIB-4 < 1.30 or 1.30 ≤ FIB-4 < 2.67 and ELF < 8.30

^b^FIB-4 ≥ 2.67 or 1.30 ≤ FIB-4 < 2.67 and ELF ≥ 9.50

^c^1.30 ≤ FIB-4 < 2.67 and 8.30 ≤ ELF < 9.50

**Fig. 1:**
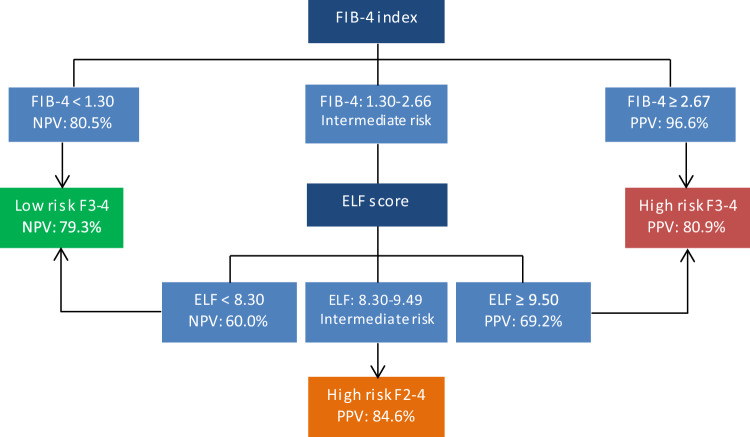
Algorithm of advanced fibrosis diagnosis in NASH cohort

The FIB-4 index alone showed the highest specificity (98%) and positive predictive value (PPV) (97%) in predicting stages F3–4 comparing with the sequential algorithm (73% and 81%, respectively). However, the sensitivity and negative predictive value (NPV) for F3–4 were the lowest in FIB-4 alone comparing with the rest of models, being of 85% and 79%, respectively, in the sequential algorithm. Furthermore, ELF alone pointed to the highest specificity (94%) in predicting stages F0–1 versus the sequential algorithm, which only reached a 71%. However, the rest of parameters were lower than the sequential algorithm, highlighting the sensitivity for F0–1 and F3–4 diagnosis. On the other hand, multivariate model achieved lower diagnostic accuracy parameters than sequential algorithm but more compensated than the FIB-4 and ELF alone. Overall PPV was 81% (high risk) and NPV was 79% (low risk), for F3–4 diagnosis in the sequential algorithm (Fig. 1). Furthermore, this algorithm pointed to high-risk significant fibrosis (F2–4) for FIB-4 and ELF intermediate values, where sensitivity and PPV were 88% and 85%, respectively. This classification was not possible when FIB-4 or ELF alone application. Finally, due to the high prevalence of T2D in the NASH cohort and its possible limitation in the FIB-4 interpretation, the accuracy of the sequential algorithm was evaluated separately between the T2D patients and non-diabetic subjects. The diagnostic performance parameters are shown in Table 4.

**Table 4:** Accuracy of sequential algorithm in the NASH patients with T2D and NASH patients without T2D

Diagnosis		Cut-off	AUC	IC95%	Sensitivity (%)	IC95%	Specificity (%)	IC95%	PPV (%)	IC95%	NPV (%)	IC95%
F0–1	With T2D	Combined^a^	0.80	0.71–0.88	75	52–90	86	74–90	52	29–76	94	85–98
	Without T2D		0.70	0.55–0.83	68	43–84	68	49–87	65	43–85	71	48–86
F3–4	With T2D	Combined^b^	0.77	0.67–0.85	89	79–97	77	60–92	91	80–97	74	54–91
	Without T2D		0.61	0.46–0.75	64	38–88	68	53–86	60	39–87	83	58–97
F2–4	With T2D	Combined^c^	0.71	0.55–0.86	88	64–99	52	31–73	88	61–99	46	25–68
	Without T2D		0.74	0.56–0.88	88	61–100	65	43–84	78	42–95	53	35–71

^a^FIB-4 < 1.30 or 1.30 ≤ FIB-4 < 2.67 and ELF < 8.30

^b^FIB-4 ≥ 2.67 or 1.30 ≤ FIB-4 < 2.67 and ELF ≥ 9.50

^c^1.30 ≤ FIB-4 < 2.67 and 8.30 ≤ ELF < 9.50

*AUC* area under curve, *PPV* predictive positive value, *NPV* negative predictive value

Additionally, FibroScan measurement was included for patients classified as high risk of F3–4 and F2–4. Ninety percent of F ≥ 2 high risk patients demonstrated a TE value ≥ 8 kPa [23]. Alternatively, TE cut-offs ≥ 9.6 kPa and ≥ 7.0 kPa were applied for F3–4 and F2–4 classifications, respectively; both cut-offs have been described previously with high diagnostic performance in NAFLD [44]. Eighty two percent of F3–4 high-risk patients demonstrated a TE value ≥ 9.6 kPa, and 96% of F2–4 high-risk patients demonstrated a TE value ≥ 7.0 kPa. The diagnostic performance parameters of F ≥ 2 diagnosis for both TE and algorithm are summarized in Table 3.

### Application of biomarkers in the primary care cohort

**Fig. 2:**
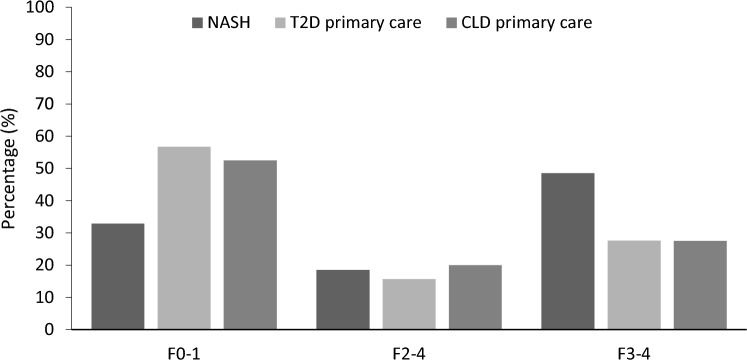
Liver fibrosis estimation of the sequential algorithm in the three study cohorts. *NASH* Non-alcoholic steatohepatitis, *T2D* Type 2 diabetes, *CLD* Chronic liver disease

The liver fibrosis diagnostic sequential algorithm was applied on the primary care cohorts of T2D and CLD to compare with the NASH cohort. In both T2D (*n* = 40) and CLD (*n* = 38) primary care cohorts, a total of 28% of patients were classified as stages F3–4. Fifty-seven percent of patients were classified as F0–1 in the T2D primary care cohort (*n* = 81) and 53% of patients were classified as F0–1 in the CLD primary care cohort (*n* = 73) (Fig. 2). Additionally, a 14% (*n* = 20) of the T2D and a 20% (*n* = 28) of the CLD cohort patients were classified as F2–4.

Regarding clinical management and diagnostic evidence of liver disease in medical records, 80% of all F3–4 classified patients in the T2D cohort had steatosis as sign of liver disease based on ultrasonography techniques, where in addition, 12% of all F3–4 classified patients in the T2D cohort had a TE value ≥ 9.6 kPa. Forty-four percent of F3–4 classified patients in the T2D cohort were referred to secondary care, such as to a gastroenterologist, endocrinologist, or hepatologist.

In the CLD cohort, 71% of F3–4 classified patients had steatosis as sign of liver disease based on sonography techniques, where in addition, 11% of all F3–4 classified patients in the CLD cohort had a TE value ≥ 9.6 kPa. Forty-two percent of F3–4 classified patients in the CLD cohort were referred to secondary care, such as to a gastroenterologist or hepatologist.

## Discussion

Our results show the need and opportunity to implement algorithms for risk stratification of liver fibrosis based on biomarkers in patients with diagnostic suspicion of NAFLD, mainly those with NASH [45, 46]. Metabolic comorbidities, i.e., T2D, obesity, and dyslipidemia are the current targets for the detection of CLD [3], to such an extent that NAFLD is considered the liver manifestation of metabolic syndrome [47]. There are likely to be varying susceptibilities to the development of fibrosis in response to a similar amount of inflammatory liver injury, but NASH is probably the main driver of fibrosis progression and is a more dynamic entity than fibrosis [34].

NASH and T2D primary care cohorts showed similar values of analytes related to glucose and lipids metabolism (Table 1), reflexing the narrow association between both identities, which occurs in bidirectional ways [48, 49]. Insulin resistance plays a key role in the pathophysiology of NASH and fibrosis development, measured by HOMA-IR calculation [50–52]. Therefore, suspicion of liver fibrosis in an IR context and other metabolic comorbidities is warranted, as occurs in the NASH cohort, where the high F3–4 prevalence coexists with the HOMA-IR suggestive of IR [53, 54].

The misdiagnosis of NASH and fibrosis after liver biopsy occurs frequently, where 23% of discordances can be attributed to a biopsy error, and the degree of fibrosis estimated by TE is greater than biopsy in 89% of those cases [55, 56]. This misdiagnosis is shown in Table 2, where the F0–1 NASH patients had a TE median value suggestive of significant fibrosis. A poor correlation between steatosis degree by ultrasonography and liver biopsy was shown too, restricting their application in the liver disease severity stratification. Thus, FIB-4 and ELF can be useful tools to screen suspicious patients of liver fibrosis, complementary to TE measurement to reinforce this diagnosis, as practiced in our NASH cohort, where TE and biomarkers values focused on the same diagnosis (Table 2). Used in this way, their optimal use can reduce the need for liver biopsy [23].

FIB-4 is a simple index composed of current routine laboratory tests, initially developed to rule out advanced fibrosis in patients coinfected with HIV/HCV [25]. It has been demonstrated that FIB-4 allows appropriate identification of NAFLD patients at a higher risk of developing liver-related complications or death [57]; currently, FIB-4 is recommended by the EASL-Lancet Liver Commission for stratification of individuals at risk of liver disease [1]. In the NASH cohort, PPV of FIB-4 index for F3–4 was 97%, being enough for high-risk advanced fibrosis classification [58]; however, to solve the low NPV (55%) when FIB-4 value < 2.67, a second step of fibrosis screening is needed.

The ELF score has been reported as a good biomarker of liver fibrosis detection [59] and it has been applied to detect NASH in obese patients with NAFLD [60]. In addition, ELF has been used to detect F2–3 [61] or F3–4 with ELF > 9.8 [62] and for F3–4 exclusion with ELF < 8.4 [29]. Also, ELF > 10.4 can predict clinical outcomes in patients with CLD [63]. Normal values are age dependent and this fact can limit predictive values when aging [61], but, because the higher sensitivity for F3–4 diagnosis than FIB-4 is observed in our results (68%), its implementation in practical assistance has been proposed as a cost-effective option compared to a single liver biopsy [64].

The algorithm proposed has higher diagnostic accuracy parameters for fibrosis diagnosis than the bivariate model (Table 3). It can be developed in one step from one blood drawn, saving physicians consultations, and it allows to guide a preliminary diagnosis from high-risk population of advanced CLD. This algorithm combines the high specificity of FIB-4 to discard F3–4 with the high sensitivity of ELF to detect F3–4 by sequential measure by combining use of two cut-off points. The limited use of individual biomarkers for the F2 detection is solved in the sequential algorithm, which achieved a high PPV (84.6%) for F2–4 when FIB-4 and ELF demonstrate intermediate values. Thus, the lower cut-off point proposed in ELF score allows F0–1 detection with high specificity (ELF < 8.30) when alone use, but also F2–4 detection (ELF ≥ 8.30) with high sensitivity in combination to FIB-4 intermediate values.

Different scenarios of liver fibrosis detection have been reported, where management in primary care or referral to specialist depends on F3-4 risk [34]. An initial FIB-4 calculation followed by ELF measurement in intermediate cases have an impact on total health care save of 25% and a reduction in hospital referrals of 70% [35].

Finally, when TE measurement is available, it can complement this biomarker-based algorithm (as performed in our NASH cohort) to reduce liver biopsy necessity. In our case, we showed a concordance of 90% of TE measurements for F ≥ 2 using the consensus cut-off ≥ 8 kPa [23, 27]. However, since the algorithm provided both an F3–4 and F2–4 high-risk classifications in separate groups, adjusting worthwhile TE cut-offs in a population at high risk for advanced liver disease, as represented by the NASH cohort studied, may provide an advantage for the degree of liver fibrosis diagnostic confirmation [65, 66]. Meanwhile, the diagnostic sensitivity of F ≥ 2 offered by the TE measurement was optimal; however, the lower specificity (30% versus 71% provided by algorithm) makes its use as a screening technique not advisable, but rather to be used in FIB-4 > 1.3 results, as the algorithm with the ELF combination measurement offers. In addition, the low availability of TE measurement limits its implementation on a large scale, so the use of biomarker-based on algorithms is more affordable, as well as stratifying in F3–4 and F2–4 high-risk different groups. So, it is worth developing safe and easily accessible noninvasive modalities to accurately diagnose NASH associated fibrosis [67].

The algorithm was proven in T2D and CLD primary care clinical settings in a pilot study. The T2D and CLD patients followed standard of care and only had a steatosis diagnosis in the majority of cases despite metabolic comorbidities—where T2D has been considered the main metabolic risk factor of advanced fibrosis [5, 49, 68]. So, liver fibrosis has to be suspicious in these patients, particularly when diagnosed with CLD [69]. Risk factor detection, such as, T2D, obesity or metabolic syndrome, is the first step in the assessment of liver fibrosis; subsequent imaging evidence of fat accumulation or liver enzyme abnormalities can trigger the non-invasive algorithm for advanced fibrosis detection [36].

Advanced fibrosis classification in both primary care cohorts was lower than in the NASH cohort, but it was 28%, a percentage high enough to consider the necessity to implement referral protocols to a secondary care specialist [23]. Since 44% and 42% of high-risk F3–4 T2D and CLD patients, respectively, were referred to a specialist and over 10% had previously a LS pathological measurement, the sequential algorithm implementation would be appropriate, because these patients would have benefited directly. This fact also reflects the necessity to set up the current referral protocols in our sanitary system to rescue the rest of high-risk patients according to noninvasive biomarkers. An estimation of the amount of ELF determinations and referrals to specialists is needed to assess the economic impact. When considering high-risk F2–4 patients, repeating biomarker-based algorithm at primary care level or TE measure would be recommended [1, 70]. Thus, the cut-offs fitting of the biomarkers allows stratify at high-risk or intermediate-risk of F3–4 and prioritize referral to specialist [66].

Guidelines have raised concerns regarding the need for community NAFLD screening because of the progressive form to NASH—particularly associated with advanced fibrosis—indicating that it should be identified in patients at risk [71]. In low-prevalence populations, noninvasive fibrosis tests should be used for ruling out advanced fibrosis, but they should be preferentially used in patients at risk of advanced liver fibrosis (such as patients with metabolic risk factors) and not in unselected general populations [27]. Additionally, it has been established that NAFLD and NASH should be suspected in patients with T2D, indicating that the clinical decision must be supported by biomarker measurement in addition to TE [2, 6, 72]. Of note, a 25% of T2D with NAFLD and FIB-4 < 1.3 who underwent liver biopsy had F3–F4 fibrosis (this percentage was 14% in patients without T2D); a FIB-4 result < 1.30 should be considered with caution in patients with T2D, and TE could help to refine the evaluation in this situation [28, 73]. In the NASH cohort studied, T2D variable inclusion in the multivariate logistic regression model did not improve the cases correctly classified (76%). Regarding the assessment of the algorithm in diabetic and non-diabetic patients separately, the diagnostic performance of F0–1 and F3–4 was higher in the T2D patients, whereas classification of the F2–4 group was more accurately done in patients without T2D. The high prevalence of F3–4 in T2D patients has been able to overcome the 25% of false negatives for FIB-4 < 1.3. On the other hand, the higher prevalence of F2 in non-diabetics (65% versus 47% in T2D subjects) can explain the improvement of the diagnostic accuracy for F2-4 in this sub-cohort.

Our study has some limitations that should be noted and that restrict the extrapolation of our results to the general population, such as: (a) the high proportion of advanced liver fibrosis in the NASH cohort could overestimate the high-risk F3–4 percentage in the primary care cohorts; (b) the absence of a gold standard method to grade liver fibrosis in the T2D and CLD primary care cohorts could limit the assessment of the algorithm concordance and direct implementation.

In conclusion, noninvasive biomarkers for liver fibrosis diagnosis allow the detection of high-risk patients with F3–4—the main outcome in CLD associated with poor prognosis in populations with metabolic risk factors. FIB-4 and ELF measurement in a sequential algorithm is a high efficiency strategy to stratify the risk of liver fibrosis in one step and prioritize patients attended at the primary care level who need specialist management and treatment.

## Data Availability

The data that support the findings of this article are available from the corresponding authors on reasonable request.
